# The Link Between Menopause and Urinary Incontinence: A Systematic Review

**DOI:** 10.7759/cureus.71260

**Published:** 2024-10-11

**Authors:** Ahmad Hasan Allafi, Amal Saleem Al-johani, Raed M Babukur, Jehad Fikri, Raneem Rashed Alanazi, Sara Dafaalla Mohamed Hag Ali, Abdulrahman Alkathiry, Abdalmelk Mohmed Alfozan, Kawthar Ishaq Ahmed Ali Husain Mayoof, Maya Ahmad Abualhamael

**Affiliations:** 1 Obstetrics and Gynecology, Maternity and Children Hospital, Arar, SAU; 2 General Practice, College of Medicine, King Abdulaziz University, Rabigh, SAU; 3 Medicine and Surgery, College of Medicine, Umm Al-Qura University, Makkah, SAU; 4 King Abdullah International Medical Research Center, College of Medicine, King Saud Bin Abdulaziz University for Health Sciences, Jeddah, SAU; 5 College of Medicine, King Saud University, Riyadh, SAU; 6 General Practice, Al-Jazeera University, Abha, SAU; 7 College of Medicine, Imam Abdurrahman Bin Faisal University, Dammam, SAU; 8 General Practice, Eastern Health Cluster, Dammam, SAU; 9 Obstetrics and Gynecology, Saudi German Hospital, Jeddah, SAU

**Keywords:** menopause, prevalence, risk factors, systematic review, urinary incontinence, women's health

## Abstract

This study aims to review the subsisting literature on the relationship between menopause and urinary incontinence (UI). To locate research that met the inclusion criteria, a thorough computerized search of relevant databases was carried out. A comprehensive search was carried out on PubMed, SCOPUS, Science Direct, Cochrane Library, and Web of Science to locate relevant material. Our data included 11 trials with 8547 post-menopausal women. The prevalence of UI among post-menopausal women ranged from 13.6% to 84.4%, with a total prevalence of 5394 (63.1%). Across studies, the mean age of menopause varies, with some studies reporting averages around 48 to 50 years. Several studies report that UI prevalence increases with age, particularly in the postmenopausal period. UI symptoms, such as burning micturition and mixed UI, are noted, often related to the genitourinary syndrome of menopause (GSM) and nocturia. These symptoms are linked to reduced quality of life and considerable morbidity. Among postmenopausal women, UI is still a common and complex problem that has a significant negative impact on well-being and life quality. There is no doubt that menopause, aging, and UI are related, but there is still much to learn about the underlying reasons and practical treatment options. The quality of care for postmenopausal women with UI can be improved by standardizing diagnostic procedures, emphasizing non-hormonal therapy, and addressing socioeconomic and lifestyle determinants. These and other initiatives will be addressed in future research and clinical practice.

## Introduction and background

Menopause, a normal physiological event, signifies the cessation of a woman's childbearing period, typically occurring between the ages of 45 and 55. During this transition, women undergo various hormonal changes, particularly a significant decline in estrogen levels [1]. This hormonal alteration not only signifies the cessation of menstrual periods but also influences several bodily functions, including those associated with the urinary system. One common and often distressing issue that emerges during or after this phase is UI, which can significantly affect a woman's quality of life [2].

UI is the involuntary loss of urine, resulting in the inability to control urination. This condition is not merely a consequence of the aging process but is exacerbated by endocrine changes associated with menopause. Estrogen plays a crucial role in maintaining the health and structural integrity of urogenital tissues, including the urinary bladder, urethra, and pelvic floor musculature [3]. As estrogen levels decline, these tissues can become atrophic and less elastic, leading to diminished pelvic support for the bladder. This physiological alteration can contribute to both stress urinary incontinence, which occurs during physical activities that exert pressure on the bladder, and urge incontinence, characterized by a sudden, compelling urge to urinate followed by involuntary urine leakage [2].

Research indicates that a significant percentage of postmenopausal women experience some form of UI. According to studies, prevalence rates can range from 25% to over 50% in this demographic, underscoring the need for increased awareness and understanding of the issue. Incontinence at this stage can be linked not only to hormonal changes but also to other factors such as weight gain, changes in lifestyle, and the presence of other medical conditions, including diabetes and urinary tract infections [4].

The impact of UI during menopause extends far beyond the physical symptoms. Many women report feelings of embarrassment, anxiety, and social isolation related to their condition. These psychological effects can lead to diminished self-esteem and reluctance to engage in social or physical activities. Notably, some women may avoid exercise, travel, or even intimate relationships due to fears of incontinence, thereby significantly affecting their overall well-being and lifestyle [5].

Fortunately, various management strategies and treatments are available for UI, and women experiencing this issue should not hesitate to seek help. Lifestyle modifications, such as weight management, pelvic floor exercises (like Kegel exercises), and bladder training can be beneficial. Additionally, healthcare providers may recommend medications that can help regulate bladder function or suggest other interventions, including physiotherapy and, in some cases, surgical procedures, depending on the severity and type of incontinence [6].

The precise nature of the association between menopause and UI remains not fully elucidated. Despite the high prevalence of UI among menopausal women, there is a lack of consensus regarding the strength and underlying pathophysiological mechanisms of this relationship. This study aims to systematically review the existing literature on the relationship between menopause and UI to provide a comprehensive understanding of how these two conditions are interconnected.

## Review

Methods

This research conducted a systematic review to investigate the association between menopause and UI, utilizing the guidelines prescribed by the Preferred Reporting Items for Systematic Reviews and Meta-Analyses (PRISMA) [7]. An electronic database search was performed to identify relevant English-language scholarly articles examining the impact of menopause on UI. The databases queried included PubMed, Web of Science, SCOPUS, and Science Direct. Keywords and medical subject headings related to UI and menopause were incorporated into the search strategy. Two reviewers independently scrutinized the search results, selected eligible studies, extracted data, and employed appropriate methodological assessment tools to evaluate the quality of the included research.

Eligibility Criteria

Inclusion Criteria: This review considers peer-reviewed research articles, including observational studies, clinical trials, and systematic reviews, which explore the link between urinary incontinence (UI) and menopause. Eligible studies must involve adult women defined as having had no menstruation for at least 12 consecutive months and should be published in English within the last 10 years. Additionally, the research must assess the frequency, significance, or effects of UI in relation to menopausal experiences.

Exclusion Criteria: Articles that are not peer-reviewed, such as editorials, commentaries, and opinion pieces, are excluded, as are studies involving populations other than adult women or those that do not specifically analyze menopause-related changes. Research focussing on unrelated urinary or pelvic floor issues as well as non-English articles will also be disregarded. Lastly, studies deemed to have a high risk of bias, poor data quality, or inadequate methodology will not be included.

Data Extraction

Rayyan was utilized to authenticate the search results, ensuring methodological precision and reliability [8]. The initial search yielded titles and abstracts, which were then appraised for relevance based on the predefined inclusion and exclusion criteria. The research team conducted a comprehensive examination of all studies that met the inclusion parameters. Any discrepancies or disagreements were resolved through consensus and scholarly deliberation. Employing a standardized data extraction form, key study variables were documented, including titles, authors, publication year, study locale, participant demographics, gender distribution, the prevalence of UI in postmenopausal women, and the association between menopause and UI. An unbiased assessment tool was developed to evaluate the potential risk of bias in the included research.

Data Synthesis Strategy

By aggregating data extracted from pertinent studies, summary tables were constructed to provide a qualitative synthesis of research findings and critical elements. Upon completing data collection for the systematic review, the most effective methodology for analyzing and utilizing the information from the included studies was determined.

Risk of Bias Assessment

The Joanna Briggs Institute (JBI) critical appraisal tool for studies reporting prevalence data was employed to assess the methodological quality of the studies [9]. This instrument comprises nine evaluative questions. A score of 1 was allocated for an affirmative response while a score of 0 was given for negative, ambiguous, or non-applicable responses. The cumulative scores were categorized to reflect study quality: scores below 4 indicated low quality, between 5 and 7 indicated moderate quality, and above 8 indicated high quality. Independent researchers appraised the quality of the papers, with any disputes resolved through scholarly discussion and consensus.

Results

Systematic Search Outcomes

**Figure 1:**
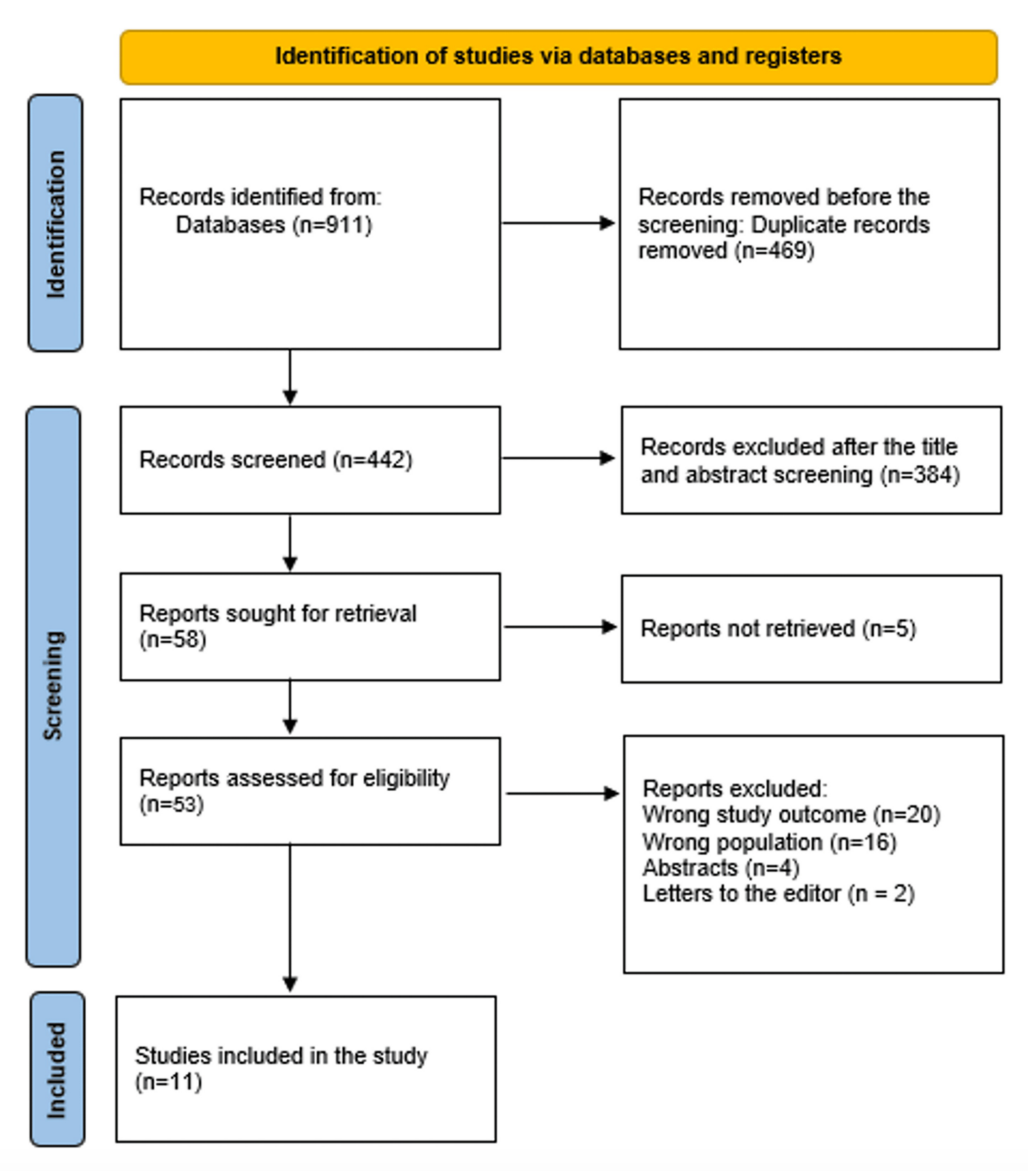
PRISMA diagram encapsulating the research decisions PRISMA: Preferred Reporting Items for Systematic Reviews and Meta-Analyses

An exhaustive search of 911 scholarly publications yielded 469 duplicate records, which were subsequently excluded. Upon critical appraisal of the titles and abstracts of the remaining 442 studies, 384 articles were deemed ineligible and rejected. Out of the 58 reports necessitated for further evaluation, 5 were unobtainable. Twenty manuscripts were excluded due to invalid or unreliable study results; two were letters to the editor, and four were abstracts without full-text availability. Sixteen of the 53 publications that passed the full-text screening stage were disqualified for using the wrong demographic types. The qualifying requirements are met by the 11 research publications that comprise this systematic review. Figure 1 illustrates the process by which the literature was searched. 

Sociodemographic and Clinical Parameters of the Comprised Participants and Studies

Table 1 summarizes the socio-demographic characteristics extracted from the research articles. Our compilation included 11 studies encompassing a total of 8,547 postmenopausal women [10-20]. The study designs comprised five cross-sectional analyses [11,15-17,19], four retrospective cohort studies [10,13,18,20], one quasi-experimental investigation [12], and one prospective cohort study [14]. Geographically, two studies were conducted in Australia [10,18], two in the United States [13,16], and one each in India [11], Egypt [12], Thailand [14], Spain [15], Japan [17], China [19], and Belgium [20].

**Table 1:** Sociodemographic parameters of the included research

Study ID	Study design	Country	Participants (n)	Mean age
Te West et al., 2024 [10]	Retrospective cohort	Australia	3546	59.9 ± 14.6
Singh et al., 2023 [11]	Cross-sectional	India	214	48.9 ± 4.7
ElSheikh et al., 2023 [12]	quasi-experimental study	Egypt	120	48.03 ± 3.04
Shardell et al., 2021 [13]	Retrospective cohort	USA	601	47
Yodplob et al., 2021 [14]	Prospective cohort	Thailand	42	66.1 ± 9.2
Moral et al., 2018 [15]	Cross-sectional	Spain	242	58.1 ± 6.9
Jones et al., 2016 [16]	Cross-sectional	USA	158	48 ± 2.2
Terauchi et al., 2015 [17]	Cross-sectional	Japan	351	52.4 ± 6.9
Trutnovsky et al., 2014 [18]	Retrospective cohort	Australia	381	56 ± 14
Zhu et al., 2022 [19]	Cross-sectional	China	2702	53.7 ± 7
Valentini et al., 2021 [20]	Retrospective cohort	Belgium	190	74.5

Table 2 shows the clinical parameters.

**Table 2:** Clinical parameters and outcomes of the comprised research Not Mentioned (NM); International Consultation on Incontinence Questionnaire (ICIQ); Lower Urinary Tract Symptoms (LUTS); Mental Health-Related Quality of Life (MHR-QOL); Urogenital Distress Inventory, Short Form (UDI-6); Genitourinary Syndrome of Menopause (GSM); Urinary Incontinence (UI); Stress Urinary Incontinence (SUI); Urge Urinary Incontinence (UUI); Urinary Stress Incontinence (USI)

Study ID	Mean age at menopause	UI diagnostic tool	Prevalence of UI (%)	Main outcomes	JBI
Te West et al., 2024 [10]	48.6 ± 5.7	Clinically	2994 (84.4%)	UI decreases as age increases.	Moderate
Singh et al., 2023 [11]	46.3 ± 1.9	Clinically	29 (13.6%)	The two most common urinary symptoms were burning micturition and UI. UI was shown to be significantly correlated	Moderate
ElSheikh et al., 2023 [12]	46.6±2.3	UIQ	78 (65%)	NM	High
Shardell et al., 2021 [13]	NM	Self-reported	200 (33.3%)	Evidence suggests that vaginal and urine microbiota are substantially concordant, however, UI may be more strongly correlated with the former than the latter.	Moderate
Yodplob et al., 2021 [14]	NM	ICIQ-LUTS	22 (52.4%)	NM	Moderate
Moral et al., 2018 [15]	NM	Clinically	83 (34.3%)	Stress and mixed UI were found to be strongly correlated with GSM of menopause.	Moderate
Jones et al., 2016 [16]	NM	Clinically	113 (71.5%)	Menopause and natural aging put women at risk for bladder issues, however, it's unclear when they are most vulnerable. UI and nocturia are two bladder ailments that are linked to considerable morbidity and reduced quality of life.	High
Terauchi et al., 2015 [17]	NM	MHR-QOL	155 (44.2%)	Significantly, LUTS is frequently observed among perimenopausal and postmenopausal women visiting menopause clinics. Frequency and nocturia are associated with non-restorative sleep; nocturia and SUI correlate with body fat or visceral fat accumulation; and UUI is linked to delayed reaction times.	Moderate
Trutnovsky et al., 2014 [18]	NM	Clinically	288 (76%)	Following menopause, hormone shortage does not appear to have a significant, age-independent impact on UUI and USI.	Moderate
Zhu et al., 2022 [19]	50 ± 3.1	UDI-6	1284 (47.5%)	Our research demonstrated that GSM is common among elderly Chinese women. GSM and UI are connected.	Low
Valentini et al., 2021 [20]	NM	Self-reported	148 (77.9%)	Regardless of age, there was a strong correlation between increased detrusor contractility and urge UI.	Moderate

The prevalence of UI among post-menopausal women ranged from 13.6% [11] to 84.4% [10], with a total prevalence of 5394 (63.1%). Across studies, the mean age of menopause varies, with some studies reporting averages around 48 to 50 years. Diagnostic tools for UI include clinical diagnoses, self-reported measures [13,20], and specific questionnaires such as Urogenital Distress Inventory, Short Form (UDI-6) [19] and the International Consultation on Incontinence Questionnaire (ICIQ)-Lower Urinary Tract Symptoms (LUTS) [14].

Several studies report that UI prevalence increases with age, particularly in the postmenopausal period [10-19]. Incontinence symptoms, such as burning micturition and mixed UI, are noted, often associated with GSM and nocturia. These symptoms are linked to reduced quality of life and considerable morbidity [11-18]. The research also highlights the socioeconomic factors contributing to the risk of UI, as well as associations with vaginal and urine microbiota [13]. Moreover, there is evidence of the connection between GSM and UI, particularly in elderly women. Interestingly, while hormone deficiency post-menopause is linked to increased UI, it doesn't seem to significantly impact UI types like UUI or USI in some studies.

Discussion

The findings of this systematic review highlight the complexity and variability of UI among postmenopausal women. The prevalence of UI among post-menopausal women ranged from 13.6% [11] to 84.4% [15], with a total prevalence of 5394 (63.1%). Robinson and Cardozo reported that the most frequent issue during menopause is UI, which affects 50% of postmenopausal women [21]. Stress incontinence constitutes the most prevalent type of urinary incontinence. Urinary incontinence is a primary symptom of GSM, adversely impacting the quality of life, daily activities, and sexual function of these women [22].

We found that across studies, the mean age of menopause varies, with some studies reporting averages around 48 to 50 years. Several studies report that UI prevalence increases with age, particularly in the postmenopausal period [10-19]. Incontinence symptoms, such as burning micturition and mixed UI, are noted, often associated with GSM and nocturia. These symptoms are linked to reduced quality of life and considerable morbidity [11-18]. Christmas et al. found that the link between menopause and symptoms related to the bladder cannot be established by the available data. HT's impact on different types of urinary symptoms varies. UI may be brought on by systemic HT, or it may exacerbate pre-existing symptoms [23].

The urogenital sinus is the embryological source of both the female genital tract and the lower urinary system. As a result, there are more estrogen receptors in the lower urinary tract and external genitalia [24]. Both estrogen and estrogen receptors decrease when menopause sets in. Being a vasoactive substance, estrogen increases these structures' vascularity and, as a result, lubrication through fluid transduction [25]. Additionally, it stimulates collagen turnover, mucin synthesis, and epithelial cell proliferation. Estrogen sustains the vaginal rugae, which aid in the vagina's distention, growth, lubrication, and adherence [15]. Thinning of the labia and vulva, reduced collagen and elasticity, reduced vascularity, vaginal atrophy, and reduced vaginal discharge are all associated with a hypoestrogenic state. Dyspareunia is brought on by the vagina's shrinking and constriction due to elasticity loss. Additionally, there is atrophy of the urethra and bladder, which results in symptoms related to the urinary system such as increased frequency and incontinence [26].

Overall, the studies emphasize the complexity of UI in postmenopausal women, where factors such as age, hormonal changes, and lifestyle contribute to the variability in prevalence and symptom severity.

Practically speaking, the results highlight how careful healthcare professionals should be when checking postmenopausal women for UI and GSM symptoms, especially in those with greater risk factors such as advanced age or particular socioeconomic situations. Early detection and treatment of UI can enhance the quality of life and lessen the impact of associated comorbidities, such as recurring UTIs or social isolation brought on by shame. Early detection and treatment may result from routine gynecological or geriatric exams that incorporate UI diagnostic instruments, such as the UDI-6 and ICIQ-LUTS. Education about non-hormonal management options, such as exercises for the pelvic floor and lifestyle modifications, may also be very beneficial, especially for women who cannot or will not receive hormone replacement therapy (HRT).

Limitations

One major limitation of the current body of research is the variability in diagnostic tools and methodologies across studies. The reliance on self-reported data in some cases may lead to under- or over-estimation of UI prevalence. Additionally, the lack of standardization in how UI is defined and measured across populations makes it difficult to compare results directly. Some studies also had small sample sizes or were limited by geographic or cultural factors, which could impact generalizability.

Furthermore, most studies fail to account for the long-term impact of various treatments on UI such as pelvic floor exercises, medications, or surgical interventions. The hormonal influence on UI remains controversial, and more controlled studies are needed to fully understand how hormone replacement therapies, or lack thereof, affect UI development in postmenopausal women.

## Conclusions

Among postmenopausal women, UI is still a common and complex problem that has a substantial negative impact on well-being and quality of life. There is no doubt that menopause, aging, and UI are related, but there is still much to learn about the underlying reasons and practical treatment options. The quality of care for postmenopausal women with UI can be improved by standardizing diagnostic procedures, emphasizing non-hormonal therapy, and addressing socioeconomic and lifestyle determinants. These and other initiatives will be addressed in future research and clinical practice. A comprehensive approach to this problem will lessen UI's negative effects on society as a whole while simultaneously improving individual health outcomes.
